# Scrotal Calcinosis: A Case Report and Review of Pathogenesis and Surgical Management

**DOI:** 10.1155/2012/475246

**Published:** 2012-07-22

**Authors:** Usman M. Tela, M. Bashir Ibrahim

**Affiliations:** ^1^Urology Unit, Department of Surgery, University of Maiduguri Teaching Hospital, Maiduguri 1414, Borno, Nigeria; ^2^Plastic and Reconstructive Unit, Department of Surgery, University of Maiduguri Teaching Hospital, Maiduguri 1414, Borno, Nigeria

## Abstract

Idiopathic scrotal calcinosis is an uncommon benign disorder of the scrotal skin which is characterized by multiple calcified intradermal nodules. We report a 33-year old with asymptomatic multiple calcified scrotal skin nodules. He had wide excision of the lesions and direct closure of the scrotum. We review the pathogenesis and surgical treatment options for this rare disease of the scrotum.

## 1. Introduction

Idiopathic scrotal calcinosis is an uncommon benign disorder of the scrotal skin characterized by multiple calcified intradermal nodules that occur in the presence of normal calcium and phosphate metabolism. This disease was first described by Lewinsky as a subtype of calcinosis cutis [1]. The pathogenesis of scrotal calcinosis is still controversial. Our aim is to report this disease in a 33-year-old man and review the pathogenesis and surgical management. 

## 2. Case Presentation 

A 33-year-old man presented to us with rashes on the scrotum of 2 years duration. The rashes have been painless, gradually increasing in size and number to the current state. There was no preceding history suggestive of sexually transmitted disease (STD), trauma, inflammation to the scrotum. He is not a known diabetic, and not on any immunosuppressive drugs. There are no features suggestive of hypercalcaemia.

**Figure 1:**
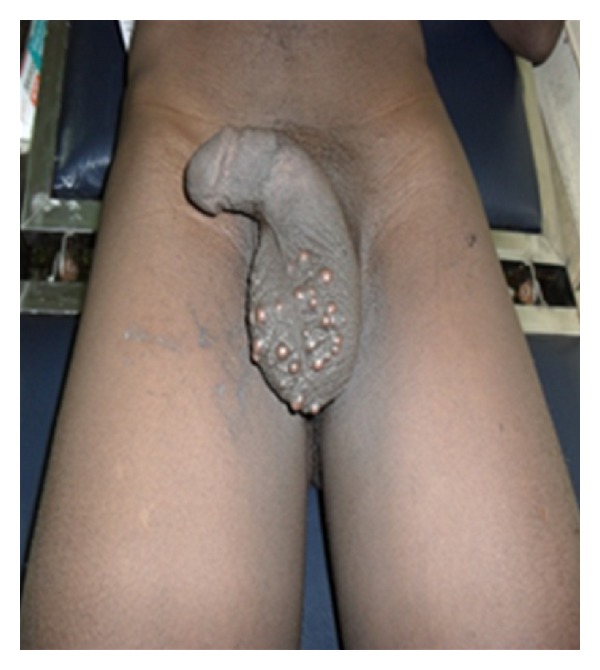
Multinodular lesions of scrotal skin.

**Figure 2:**
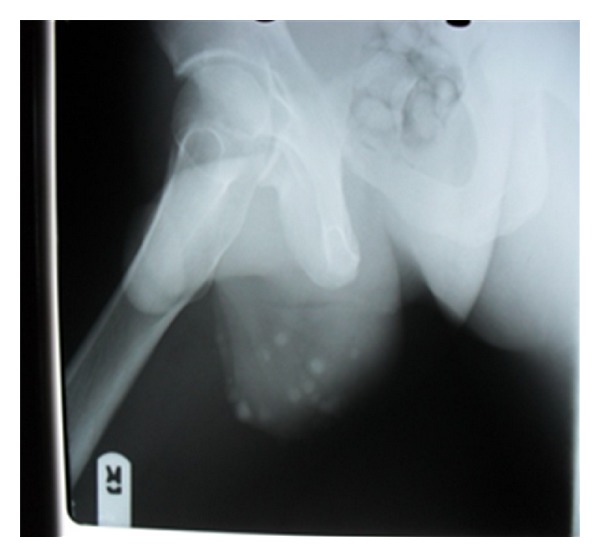
X-ray showing calcific scrotal nodules.

On physical examination, he was fit looking with Athletic physique. Review of systems was normal. The main finding was on scrotal examination, which revealed multiple nodular lesions involving the ventral surface of the scrotum, sparing other part of the scrotum and the penis. The largest nodule measured about 6 mm by 5 mm (Figure 1). The lesions were not ulcerated or tender. Scrotal X-ray revealed multiple opacities in area of the lesions (Figure 2). Serum calcium, phosphate and albumin were within reference value. Diabetes and retroviral screening were negative. 

**Figure 3:**
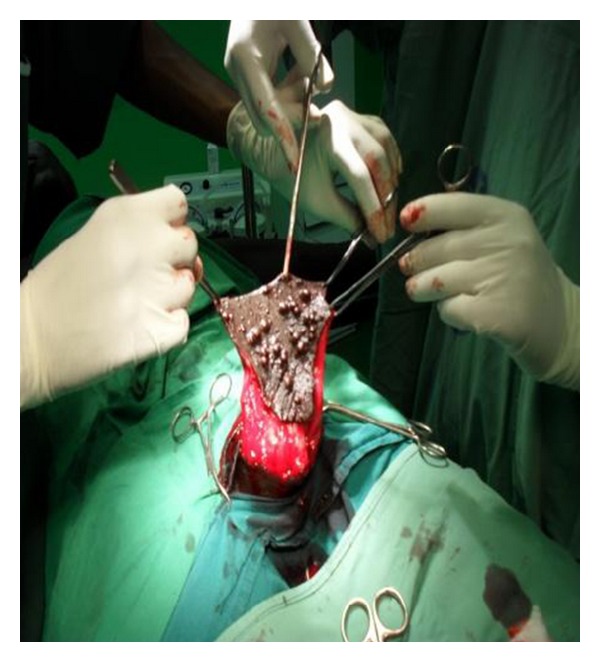
Excision of scrotal calcinosis.

**Figure 4:**
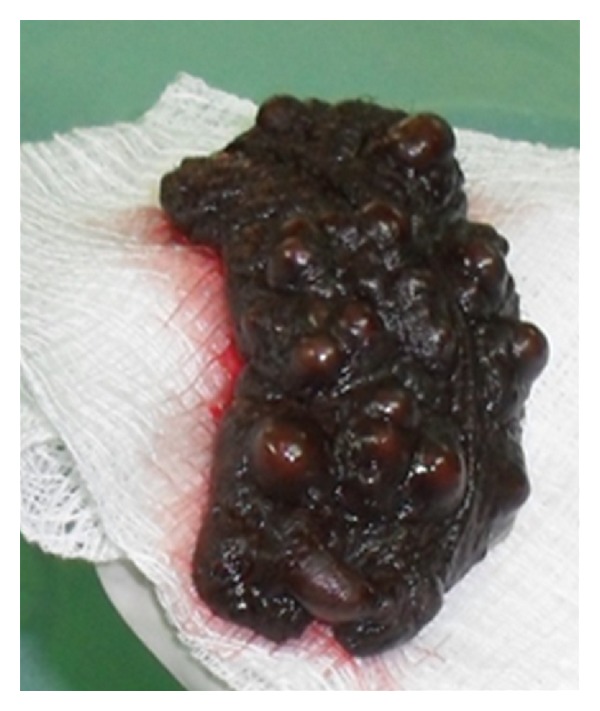
Postexcision scrotal calcinosis spacemen.

**Figure 5:**
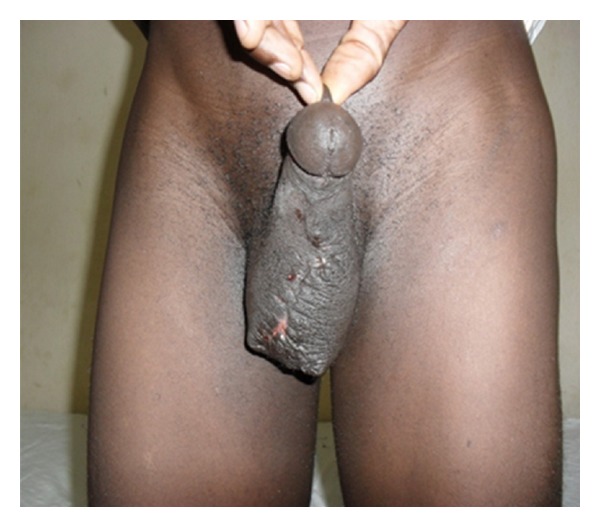
Two weeks postoperative.

Histology of the incisional biopsy showed calcium deposits in the dermis of scrotum surrounded by pseudocapsule and histiocytic inflammation. No evidence of cyst wall or keratin. He requested for excision on cosmetic ground. Wide local excision of lesion with direct closure was done with good postoperative outcome. The intraoperative and postoperative findings were shown in Figures 3, 4, and 5. Histology of the excised lesion remained the same. He was seen 16 months postoperatively, with no evidence of recurrence.

## 3. Discussion 

Scrotal calcinosis is characterized by calcific deposits with surrounding foreign bodytype granulomatous inflammation in the scrotal skin. This benign scrotal lesion, though commonly occurs between third and fourth decades of life, can affect both adult and paediatric age groups with age range between 9 to 85 years reported in the literature [2]. Scrotal calcinosis is more common in dark coloured race [3] and affects mainly male but similar lesions (vulvar calcinosis) has been reported in female [4]. 

Although Hicheri et al. reported rapidly evolving variant which occurred within 3 months [5]; the disease usually takes an indolent course, developing over several years as shown by the index case. Most patients are asymptomatic and present because of cosmetic concern. Few patients may present with pruritus, ulcerations, and discharge of chalky material with occasional superimposed secondary bacterial infection. Clinical diagnostic confusion may arise from other scrotal lesions such as calcified onchocercoma [6], solitary neurofibromas, ancient schwannomas, steatomas, lipoma, and fibroma. Biopsy for histological examination is necessary to differentiates scrotal calcinosis from such lesions. In scrotal calcinosis amorphous basophilic calcium deposits surrounded by monocytic or histiocytic inflammation can be seen on histological examination. 

Pathogenesis still remains elusive and continues to be debated. Generally, extraskeletal calcifications are classified into idiopathic, dystrophic, or metastatic calcifications. Scrotal calcinosis occurs in the absent of calcium and phosphate metabolic abnormalities. The bone of contention is whether the calcification is triggered by presence of pathological lesion (dystrophic calcification) or occurs in normal scrotal tissue in the absent of a known underlying pathological process (idiopathic calcification).

Many authors proposed that dystrophic calcification of preexisting lesion like epidermal cyst [1, 7–9], eccrine duct milia [10], degenerated dartos muscle as the underlying aetiopathogenesis of this disease. Dubey et al. suggest that inflammation of epidermal cyst leads to calcification of the cyst wall; with subsequent degeneration of cyst wall living only the calcific deposits in older lesions [9]. Dare and Axelsen using immunohistochemistry and CEA antibodies demonstrated the involvement of eccrine duct milia in scrotal calcinosis [10]. He proposed the term hydra calcinosis of the scrotal skin. 

Carson highlights the possible role of nanobacteria in extraskeletal calcifications [11]. They can invade the skin via the sites of microtrauma without causing overt features of infection. Their most remarkable characteristic is the formation of calcium apatite crystal at neural PH and at physiologic level of blood calcium and phosphate [11]. Our index case and many other reports failed to demonstrate presence of cyst wall or keratin around the lesion apart from the fibrous pseudocapsule [12, 13]. This type can be referred to as idiopathic. 

The main reason patient seek intervention is because of cosmetic concern. Patient with intense pruritus or ulceration will require surgical intervention. Smaller lesions are amenable to the novel pinch punch excision [14]. Larger lesions may require wide excision and direct closure can be achieved in most patients as shown in our index case. Extensive disease involving the whole scrotum or florid recurrent disease will require complex scrotal reconstruction. 

Scrotal skin has unique cosmetic and functional features that make reconstruction difficult. Ruggal nature and thinness of the testicular covering is important for temperature control and optimal spermatogenesis. Mesh skin graft provide a thin covering and a design similar to ruggal skin. Skin flap from the groin or medial circumflex femoral perforator flap can provide thin and mobile cover for scrotal reconstruction. Demir and coworkers using Johnsen score for spermatogenesis found that use of graft in animal for scrotal reconstruction diminishes testicular function whereas use of flaps resulted in testicular function similar to control group [15]. More human study needs to be carried out on the best option for scrotal reconstruction.

Even though scrotal calcinosis is a benign condition, it is important to let patient know about the possibility of recurrence [8]. Recurrence may be due to left over microscopic foci of calcification.

## 4. Conclusion 

Scrotal calcinosis is an uncommon disease with multiple scrotal nodules which is amenable to excision and direct closure but occasionally may require complex scrotal reconstruction.
